# Dual EGFR and mTOR targeting in squamous cell carcinoma models, and development of early markers of efficacy

**DOI:** 10.1038/sj.bjc.6603656

**Published:** 2007-03-06

**Authors:** A Jimeno, P Kulesza, J Wheelhouse, A Chan, X Zhang, E Kincaid, R Chen, D P Clark, A Forastiere, M Hidalgo

**Affiliations:** 1 Sidney Kimmel Comprehensive Cancer Center, The Johns Hopkins University School of Medicine, 1650 Orleans Street, Room 1M89, Baltimore, MD 21231-1000, USA; 2 Department of Pathology, The Johns Hopkins University School of Medicine, 1650 Orleans Street, Room 1M89, Baltimore, MD 21231-1000, USA

**Keywords:** dual targeting, erlotinib, temsirolimus, EGFR, mTOR, squamous cell carcinoma

## Abstract

The epidermal growth factor receptor (EGFR) is a validated target in squamous cell carcinoma (SCC) of the head and neck. Most patients, however, do not respond or develop resistance to this agent. Mammalian target of rapamycin (mTOR) is involved in the pathogenesis of SCC of the head and neck (SCCHN). This study aimed to determine if targeting mTOR in combination with EGFR is effective in SCC, and to develop early pharmacodynamic markers of efficacy. Two SCC cell lines, one resistant (HEP2) and one of intermediate susceptibility (Detroit 562) to EGFR inhibitors, were xenografted *in vivo* and treated with an mTOR inhibitor (temsirolimus), an EGFR inhibitor (erlotinib) or a combination of both. Temsirolimus exerted superior growth arrest in both cell lines than erlotinib. The combined treatment resulted in synergistic antitumor effects in the Detroit 562 cell line. Immunohistochemical assessment of pharmacodynamic effects in fine-needle aspiration (FNA) biopsies early after treatment using phospho MAPK, Phospho-P70 and Ki67 as end points demonstrated pathway abrogation in the Detroit 562 tumours treated with the combination, the only group where regressions were seen. In conclusion, an mTOR inhibitor showed antitumor activity in EGFR-resistant SCC cell lines. Marked antitumor effects were associated with dual pathway inhibition, which were detected by early FNA biopsies.

Squamous cell carcinoma (SCC) of the head and neck (SCCHN) is the sixth most common cancer in the developed world, affecting nearly 44 000 patients and causing 11 000 deaths each year in the US alone (Jemal *et al*, 2006). The epidermal growth factor receptor (EGFR) is a validated target in SCC of the head and neck (Bonner *et al*, 2006). Erlotinib (Tarceva; OSI Pharmaceuticals, Uniondale, NY, USA) and gefitinib (Iressa; Astrazeneca Inc., Willmington, DL, USA) are quinazoline derivatives that reversibly inhibit the tyrosine kinase (TK) of EGFR, showing *in vitro* and *in vivo* activity in SCCHN cell lines (Amador *et al*, 2004; Jimeno *et al*, 2006) as well as in phase II clinical trials (Soulieres *et al*, 2004; Kirby *et al*, 2006). Tumour responses induced by these agents are, however, infrequent and transient and even sensitive patients rapidly develop secondary resistance. Thus, developing therapeutic alternatives for such patients is a necessity. The predominant mechanism underlying resistance to these agents is unknown. Failure of one single strategy to fully inhibit the pathway has been suggested and, in fact, dual targeting of the EGFR results in improved antitumor effects in preclinical models of SCC of the head and neck (Huang *et al*, 2004; Matar *et al*, 2004; Jimeno *et al*, 2005). Another hypothesis is that persistent activation of downstream pathways abrogates the effects of the agents. Thus, one attractive approach is to combine EGFR inhibitors with other signalling inhibitors targeting downstream pathways.

It has recently been shown that SCC have elevated levels of Akt activation (Amornphimoltham *et al*, 2004). In other preclinical models such persistent activation of Akt has been linked to resistance to EGFR inhibitors (Bianco *et al*, 2003). Furthermore, a substantial body of preclinical studies suggest that tumours with persistent Akt activation have heightened responses to inhibition of the mammalian target of rapamycin (mTOR) (Neshat *et al*, 2001; DeGraffenried *et al*, 2004; Gera *et al*, 2004). A recent study in SCC cell lines demonstrates that inhibition of mTOR induces activation of Akt and results in antitumor effects in this tumour type (Amornphimoltham *et al*, 2005). In this model, as well as in other disease types such as pancreas cancer (Bruns *et al*, 2004), the antitumor effects of mTOR inhibitors appear to be in part related to their antiangiogenic effects. In our previous work, we have determined the susceptibility of a set of SCC cell lines both *in vitro* and *in vivo* to EGFR inhibitors (Amador *et al*, 2004; Jimeno *et al*, 2006). In the current work, we tested whether mTOR inhibition with temsirolimus (Wyeth, Madison, NJ, USA) alone and in combination with erlotinib would be efficacious in those EGFR-resistant SCC *in vivo* models. We investigated the utility of fine-needle aspiration (FNA)-based early assessment of pharmacodynamic response by immunohistochemistry assessing both proximal Phospho-MAPK (pMAPK) and phospho-P70 (pP70) and distal (Ki67) markers. These early biopsies were also used to investigate changes in the expression profile of relevant genes potentially related to angiogenesis and efficacy of EGFR and mTOR inhibitors.

## MATERIALS AND METHODS

### Drugs

Erlotinib was provided by OSI Pharmaceuticals (Uniondale, NY, USA). Temsirolimus was provided by Wyeth (Madison, NJ, USA).

### Cell lines and *in vitro* culture conditions

The cell lines used in this study (HEP2 and Detroit 562) were obtained from the American Type Culture Collection (Manassas, VA, USA), and are derived from cervical (HeLa contaminant) and head and neck squamous carcinomas, respectively. These cell lines were used based on our prior knowledge of their sensitivity to erlotinib as well as the existing information with mTOR inhibitors in the model. The cell lines were grown in six-well plates with RPMI media supplemented with 10% fetal bovine serum (FBS) and 1% penicillin/streptomycin (growth media (GM). When 50–60% confluency was reached, they were serum starved overnight, after which they were exposed to (1) serum-starved media for 2 h and 30 min, (2) serum-starved media for 30 min followed by GM for 2 h, (3) serum-starved media plus erlotinib (10 *μ*M) for 30 min followed by GM plus erlotinib for 2 h, (4) serum-starved media plus temsirolimus (10 *μ*M) for 30 min followed by GM plus temsirolimus for 2 h and (5) serum-starved media plus the combination of erlotinib (10 *μ*M) plus temsirolimus (10 *μ*M) for 30 min followed by GM plus erlotinib plus temsirolimus for 2 h. After this the cells were washed twice with chilled PBS, lysis buffer was added to the plates and the cells were scraped for protein extraction. An identical experimental set was lysed directly from the tissue culture dish with RLT (Qiagen, Valencia, CA, USA) for RNA extraction.

### *In vivo* growth inhibition studies

Six-week-old female athymic nude mice (Harlan, IN, USA) were used. 2 × 10^6^ HEP2 and Detroit 562 cells were injected subcutaneously in each flank. Tumours were allowed to grow until reaching 200 mm^3^, at which time mice were randomized in the following four groups of treatment, with five to six mice (10 evaluable tumours) in each group: (1) control, (2) erlotinib 50 mg kg day^−1^ i.p., (3) temsirolimus 20 mg kg day^−1^ i.p., and (4) erlotinib plus temsirolimus at the above doses. Treatment was given for 28 days. Mice were monitored daily for signs of toxicity and were weighed three times per week. Tumour size were evaluated two times per week by caliper measurements using the following formula: tumour volume=(length × width^2^)/2. Relative tumour growth inhibition was calculated by relative tumour growth of treated mice divided by relative tumour growth of control mice (T/C).

### FNA biopsies

Fine needle aspirations (FNAs) on mice were performed according to standard cytopathologic practice under inhaled general anaesthesia (isofluorane) using 10 cm^3^ syringes and 25-gauge needles. During each FNA procedure, the first pass was smeared onto glass slides and used for morphologic analysis, (DiffQuik™ and Papanicoloau), the second and third passes were harvested in RLT buffer for RNA analysis, and the fourth and fifth passes in formalin for immunohistochemical purposes. Six tumours per treatment arm were biopsied at baseline and after 7 days of therapy.

### Western blot analysis

Equal amounts of protein were resolved on 10% polyacrylamide gels. Gels were transferred onto nitrocellulose membranes that were incubated overnight at 4°C with antibodies against phospho-EGFR, total EGFR, pMAPK, total MAPK, phospho-Akt, total Akt, pP70, total P70 (Cell Signaling Technology, Beverley, MA, USA) and actin (Santa Cruz Biotechnology, Santa Cruz, CA, USA). The immunoreactive proteins were detected using the enhanced chemiluminescence method (Amersham, Piscataway, NJ, USA).

### RNA extraction

For RNA extraction from the FNA samples, two passes from the FNA were put in RLT lysis buffer (Mini RNeasy, Qiagen) and total RNA was extracted using the Rneasy™ Mini Kit (Qiagen). RNA was transcribed into cDNA by reverse transcription by priming with random hexamers (M-MLTV, Promega, Madison, WI, USA). The excess hexamers were removed using a column-based clean-up kit (Qiagen).

### Quantitative real-time RT–PCR analysis

Samples were analysed in a blinded manner. For c-fos, egfr and HIF-1alpha determination on *in vitro* samples and FNAs from mice tumours, quantitative PCR was performed on an MX3000p thermal cycler (Stratagene, Lajolla, CA, USA) using SYBR green dye method to track the progress of the reactions with ROX dye added as reference. Beta-Globin DNA specific primers were used to test DNA contamination for each sample type. Three housekeeping genes (HPRT, UBC and SDHA) were run in parallel with test genes. The amount of change in the target gene between the control and experimental conditions was found by comparing the threshold cycle (Ct) of the target gene to the geometric mean of the Ct's of the housekeeping genes. The geometric mean of the Ct's of each of the housekeeping genes, and a change in Ct (delta Ct), between conditions were calculated (dCt_Houskeeping_=(Ct_HPRT_^*^Ct_UBC_^*^Ct_SDHA_)_control_−(Ct_HPRT_^*^Ct_UBC_^*^Ct_SDHA_)_exp_). The change in Ct for the target gene was calculated directly from Ct under each condition (dCt_target_=(Ct_target_)_control_−(Ct_target_)_exp_). The efficiency of the housekeeping genes raised to their dCt divided by the efficiency of the target gene raised to its dCt gave a ratio between the control and experimental conditions normalised to the housekeeping genes (Ratio=*E*_Target_^dCtTarget^/*E*_Housekeeping_^dCtHousekeeping^, where *E* is the Primer efficiency and Ct is the Threshold cycle).

### Immunohistochemical analysis

The pathology team was blinded for treatment allocation and efficacy of the samples. Five-micrometre formalin-fixed paraffin cuts were used for Ki67 staining that was performed following the manufacturer's instructions (08-0156; Invitrogen, Carlsbad, CA, USA), and scored as percentage staining nuclei. Phospho-Thr202/Tyr204 MAPK (4376; Cell signaling Technology, Beverly, MA, USA) and phospho-p70/S6K (9206; Cell signaling) staining was performed using citrate-steam recovery, followed by Catalyzed Signal Amplification II biotin-free tyramide kit (DAKO, Carpinteria, CA, USA). Both the intensity (0, 1+, 2+, 3+) and the percentage (0–100%) of cells positive were considered. Three tumours per treatment group were analysed. For statistical analyses, an index of intensity × percentage was calculated. CD31 staining (550274; BD Pharmingen) was performed on zinc-fixed tissue (51-7538KZ; BD Pharmigen). Two methods for vessel quantification were used. First, microvessel density (MVD) was scored according to the standard methods (Weidner *et al*, 1991). Briefly, four areas per tumour were chosen and microvessel per high-power field (HPF; × 200) were counted. Second, we developed a novel scoring system based on quantifying mouse vessel invasion of the xenografts from the capsule. This was scored according to the following: (1) Capillaries in the pseudocapsule only, surrounding the tumour less than entire circumference. (2) Capillaries in the pseudocapsule only, surrounding the entire circumference of the tumour. (3) Capillaries in less than 50% of the deeper stroma, but not extending to the tumour centre. (4) Capillaries in more than 50% of the deeper stroma, but not extending to the tumour centre. (5): Capillaries throughout the tumour, including the centre. Six tumours per treatment group were analysed.

### Statistics

The comparisons between means and proportions obtained from the biologic studies were carried out using Student's *t*-test and *χ*^2^ method, respectively.

## RESULTS

### Pathway modulation analysis

**Figure 1:**
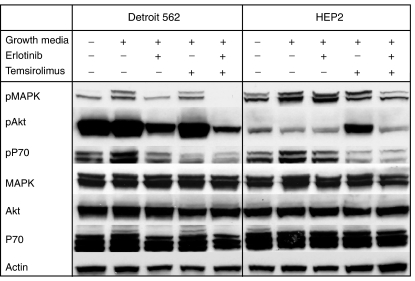
Analysis of the pathway activation profile after exposure to the study drugs. Cells were serum-starved overnight, after which they were preincubated with erlotinib (10 *μ*M), temsirolimus (10 *μ*M) and the combination of both (10 *μ*M) followed by GM containing the same drugs. The experiment was controlled by a continuously serum starved group, and a group that received GM without preincubation with any drug. In Detroit 562 erlotinib was able to decrease pMAPK, although only the combination abrogated its activity completely. On the other hand, the effect in HEP2 was much less apparent. Phospho-Akt was significantly abrogated in Detroit 562 by erlotinib-containing arms (particularly the combination), but not by temsirolimus, indicating a more downstream level of inhibition. On the other hand, in HEP2 cells lower levels of pAkt were observed at baseline, no significant increase was seen upon exposure with GM, and no inhibition was seen after erlotinib treatment. However, a clear upregulation was observed when treated by temsirolimus alone; even more notably, this upregulation was not documented when both erlotinib and temsirolimus were combined. Inhibition of pP70 was seen in Detroit 562 and HEP2 cells after erlotinib and more markedly after temsirolimus or the combination.

We first profiled the selected cells lines *in vitro* to determine the impact of the drugs in the activation of signalling pathways in this model (Figure 1). In Detroit 562, erlotinib alone inhibited MAPK activation and reduced the activation of Akt and P70. Temsirolimus predominantly inhibited P70 and induced no effects in the phosphorylation of MAPK and/or Akt. The combined treatment completely abrogated pMAPK, and significantly inhibited Akt phosphorylation. HEP2 responded similarly but the degree of inhibition was lower. Erlotinib alone had minimal effects in the signalling pathways assessed; temsirolimus, however, induced Akt activation and blocked P70, and the combination exerted increased inhibition of pMAPK with a clear abrogation of temsirolimus-induced Akt activation in this cell line.

### *In vivo* growth inhibition analysis

**Figure 2:**
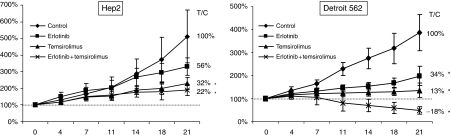
*In vivo* growth of HEP2 and Detroit 562 cell lines xenografted in nude mice treated with vehicle (♦), erlotinib (50 mg kg day^−1^ i.p.; ▪), temsirolimus (20 mg kg day^−1^ i.p.; ▴) and a combination of both (same doses; ×). The growth of the control group since the day treatment started to day 21 of the experiment was set as the reference (100%). In HEP2 xenograts erlotinib alone did not delay growth significantly, reaching 56% of the control volume by 21 days. Temsirolimus did decrease growth to 32%, and the combination to erlotinib and temsirolimus induce a growth arrest of 22% of the control group. However, the differences between the latter two groups were not significant. In Detroit 562, erlotinib alone delayed growth to 34% of the control volume by 21 days. Temsirolimus slowed growth to 13%, and the combination actually induced tumour regressions. Error bars represent s.d. (*n*=10 tumours per group). ^*^P⩽0.05 compared to control.

The *in vivo* data are summarised in Figure 2. In HEP2, considering the growth of the control group from treatment commencement to the end of the experiment after 21 days as a 100% growth, erlotinib alone did not delay growth significantly, reaching 56% of the control volume by 21 days (Figure 2A). Temsirolimus slowed growth to 32%, and the combination to 22% of the control group. This represents an average tumour size of 230 and 191% compared to the initiation of the experiment for the latter two treatment groups, respectively.

In Detroit 562, erlotinib alone delayed growth to 34% of the control volume by 21 days (Figure 2A), which corresponds to a 198% growth from the start of the trial. Temsirolimus slowed growth to 13%, and the combination achieved a −18% of the control group, representing a final tumour size of 136% for temsirolimus and a decrease to 49% of the initial volume in the combination arm (the only where actual tumour regressions were seen).

### Tumour immunohistochemical analysis

**Figure 3:**
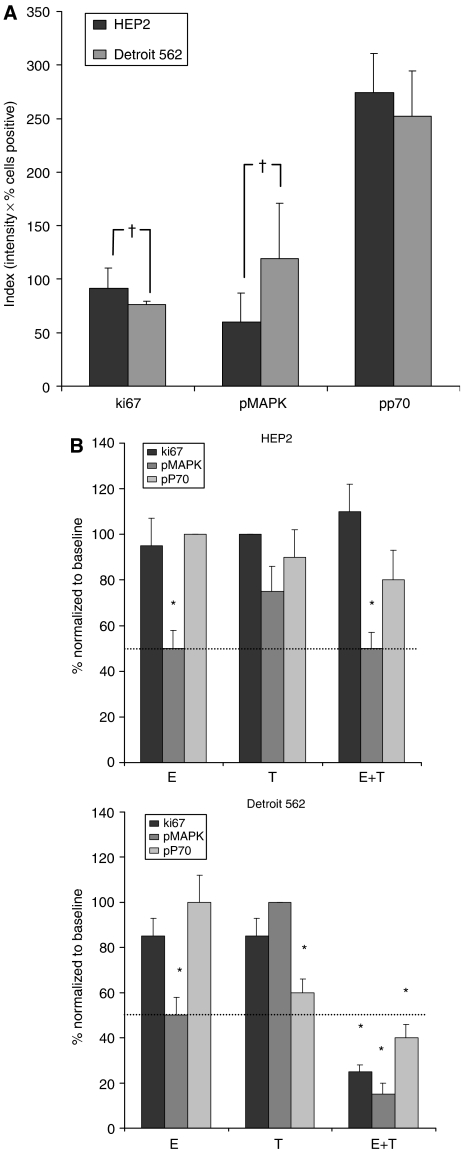

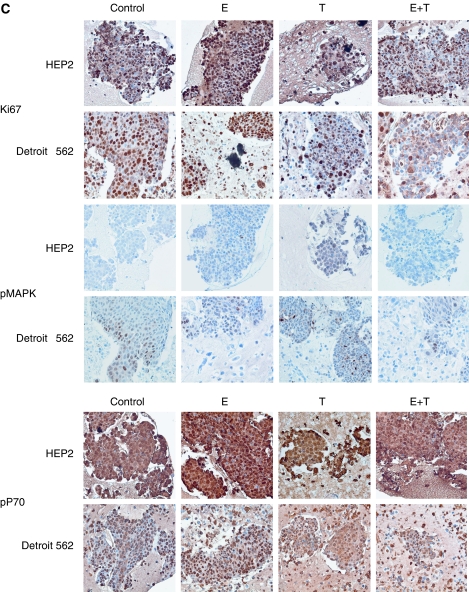
(**A**) The baseline levels of tumour Ki67, pMAPK and pP70 were averaged and compared with Student's *t*-test. Proliferation as assessed by Ki67 was higher in HEP2 and pMAPK was higher in Detroit 562. (**B** and **C**) Analysis and microphotographs of FNA cores. Variation in Ki67, pMAPK and pP70 levels were assessed by means of sequential FNAs of tumours at baseline and after 7 days of therapy. Each day 7 value was normalised in percentage to its baseline value. Values in the *Y* axis represent percentage change compared to baseline. Phospho mitogen-activated protein kinase decreased in all four arms treated with erlotinib; in Detroit 562 the IHC changes were similar to those observed in the Figure 1 Western blots. However, in HEP2 the signalling inhibition was less evident *in vivo* than *in vitro*, as little pP70 inhibition was documented. Indeed, pP70 only decreased significantly in Detroit 562 tumours. The only group where a significant (⩾50%; cutoff arbitrarily selected) decrease in all three markers was observed corresponded to the combination arm of Detroit 562. ^†^*P*⩽0.05. ^*^P⩽0.05 compared to baseline control.

In each xenograft model, 16 tumours (four per treatment arm) were followed by FNA at baseline and on day 7 of treatment for immunohistochemical analysis. The results are shown in Figure 3. In the comparison of the baseline FNA samples, the proliferation index Ki67 was higher in HEP2 than in Detroit 562 tumours (91 *vs* 74%, *P*=0.041), whereas the activation status index (intensity × percentage) of MAPK was higher in Detroit 562 tumours (60 *vs* 119, *P*=0.018). This was related to a higher percentage of cells staining positive (24 *vs* 49%, *P*=0.038), as the intensity in both groups was similar. Phospho-P70 staining index was high in both cell lines and showed no differences (274 *vs* 253, *P*=0.31) (Figure 3A).

On day 7 after treatment, neither Ki67 nor p-MAPK showed a decrement in the HEP2 cell line. Phospho-P70 decreased mildly (15 and 25%) after single-agent temsirolimus and the combined regimen, respectively. In Detroit 562 tumours, Ki67 and pMAPK only decreased significantly (>50%) after combination therapy. Phospho-P70 decreased after single-agent temsirolimus but more dramatically with the combined therapy (40 and 60%, respectively) (Figure 3B and C).

Therefore, FNA on day 7 was able to predict in both groups the activity (or lack of) of the different agents by day 21. A significant decrease in all three markers was only achieved in the combination arm in the Detroit 562 cell line, and it was the only arm where actual tumour regression was observed. Tumour stability (observed in Detroit 562 treated with temsirolimus, and in HEP2 treated with temsirolimus and the combination) was heralded by modest (<50%) decreases in pP70 staining index.

### Tumour gene expression analysis

In each xenografted cell line, 24 tumours (six per treatment arm) were followed by FNA at baseline and on day 7 of treatment for gene expression analysis. The gene expression RT–PCR analysis of the baseline FNA samples showed that HIF-1*α* mRNA expression was nonsignificantly lower in HEP2 than in Detroit 562 tumours (7.9 *vs* 15.1 (arbitrary units), *P*=0.064), whereas there was no difference in c-fos (432 *vs* 708, *P*=0.39) or EGFR mRNA (6.4 *vs* 8.5, *P*=0.41). When days 0 and 7 were compared, HIF-1*α* mRNA increased two- and fourfold in HEP2 and Detroit 562 control tumours, respectively. *C-fos* levels increased twofold in Detroit 562 control tumours, whereas no significant change was documented in c-fos in HEP2, or in EGFR mRNA levels in either of the two models. In HEP2 tumours, HIF-1*α* increased after treatment with erlotinib, did not change with temsirolimus and decreased three-fold in the combined arm. In Detroit 562, HIF-1*α* did not change with any single agent, but decreased eight-fold in the combined arm. As previously described in similar models (Jimeno *et al*, 2006), *c-fos* levels were not suppressed by erlotinib in either HEP2 or Detroit 562 xenografts (data not shown). Epidermal growth factor receptor mRNA levels increased in response to erlotinib in the resistant HEP2, whereas did not change in the intermediately sensitive Detroit 562, in line with previous reports (data not shown) (Jimeno *et al*, 2005).

### Angiogenesis evaluation

**Figure 4:**
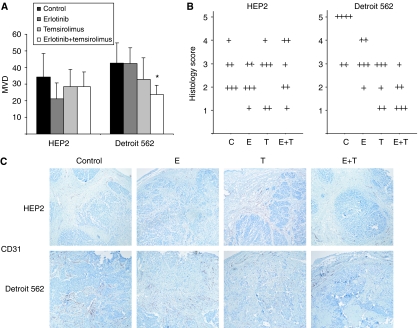
(**A**) MVD was blindly scored on four areas in each of three tumours per group. In HEP2 no significant changes were seen. In Detroit 562, the tumours treated with the combination had 24 *vs* 43 vessels on average (*P*=0.068). (**B**) Vessel in-growth assessment. Tumours were blindly scored 1–5 depending on the extent of vessel invasion inside the tumour. Each individual tumour is represented by a ‘+’ symbol. In HEP2 there were no significant differences between treatment groups. In Detroit 562 tumours, a higher density of vessels was evidenced than in HEP2. Most of the control tumours in both cell lines had vessels reaching the centre of the tumour. In Detroit 562 the temsirolimus and to a greater extent the combined arm had a great decrement in vessel intratumor growth. Half of the tumours in the latter group showed no evidence of vessel invasion from the capsule inside the tumour, and none had vessels reaching the centre. (**C**) Representative images from the above groups. Error bars indicate s.d. C: control; E: erlotinib; T: temsirolimus. ^*^P⩽0.05 compared to control.

Tumours were blindly examined for vessels by CD31 staining. The standard MVD showed no significant changes between treatment groups in HEP2 (Figure 4A). In Detroit 562 there was a higher density of vessels than in HEP2. Single-agent erlotinib or temsirolimus administration failed to induce any significant changes in MVD. However the tumours treated with the combination presented a trend towards lower MVD count compared with control tumours (24 *vs* 43 vessels/HPF, respectively; *P*=0.068). Then we implemented a novel scoring system that accounted for the pattern of microvessel invasion in the tumours. In HEP2 there were again no significant differences between treatment groups, as depicted in Figure 4B and C. All Detroit 562 tumours assessed presented vessels in the periphery/pesudocapsule of the tumour; however, the intratumor growth varied significantly between treatment groups. Most of the control tumours had widespread vessels reaching the centre of the tumour. However, the temsirolimus but more importantly the erlotinib plus temsirolimus group had a decrement in vessel intratumor growth. Half of the tumours in the latter group showed no evidence of vessel budding from the capsule inside the tumour, and none of the tumours treated with the combination had vessels reaching the centre.

## DISCUSSION

There is increasing interest in combining signalling inhibitors in cancer treatment. This study explored if combining treatment with an EGFR and an mTOR inhibitor in an SCC model with intermediate susceptibility to EGFR inhibition. The results show that an mTOR inhibitor alone was superior to an EGFR inhibitor in this model. In addition, the combined treatment was clearly superior in one cell line but not the other with a profound tumour regression elicited in the Detroit 562 line. Fine-needle aspiration aspirate tumour biopsies performed on day 7 after treatment showed greater pathway and angiogenesis modulation in the combined treatment that accurately predicted the tumour growth inhibition observed on day 21.

The analysis of pathway activation showed an upstream activation in Akt induced by mTOR inhibition in the EGFR-resistant cell line; other groups have previously described this phenomenon (O'Reilly *et al*, 2006). Those authors suggest that cells with constitutive activation of mTOR have a feedback downregulation of receptor TK signalling leading to Akt inactivation. Reversal of this feedback loop by mTOR induces Akt activation. In a separate work, two transcripts mediating mTOR expression were identified, cyclin D1 and c-myc, which exhibited differential expression in an Akt-dependent manner: high levels of activated Akt resulted in rapamycin-induced downregulation of expression, whereas low levels resulted in upregulation of expression (Gera *et al*, 2004). The current report is concordant with the notion that activation of Akt upon mTOR inhibition is a mechanism of resistance to these agents as the HEP2 cell line in which this phenomenon was observed was clearly resistant. As expected, the two agents combined blunted the activation of Akt induced by temsirolimus. Interestingly, in the cell line where erlotinib was capable of abrogating Akt activity (Detroit 562), an at least additive effect was observed with dual treatment; on the other hand, in HEP2 where erlotinib had no effect on Akt the combined treatment was equally effective as the best single agent. It can be hypothesised that the ability of erlotinib to decrease the level of activation of Akt is important for antitumor effect to occur.

A relevant aspect of this work is the demonstration of the value of FNA-based pharmacodynamic assessment for the prediction of efficacy. Fine-needle aspiration early in therapy was able to predict in both groups the activity (or lack thereof) of the different agents after a conventional treatment cycle; a significant decrease in all three markers was only achieved in the sole treatment arm where actual tumour regression was observed. Inhibition of pMAPK was consistently seen in all four erlotinib-treated groups, but did not correlate with growth inhibitory effect. Globally, this may indicate that the sole assessment of a proximal marker may be inefficient to prognosticate outcome, and combinations of several proximal and distal markers may render better prediction results. In this work we examined two pathway-specific proximal markers (phospho-MAPK and phospho-P70) and a nonspecific distal marker (Ki67) in order to comprehensively assess drug effect. We have previously reported that proximal target inhibition is necessary but not sufficient to achieve antitumor effect (Jimeno *et al*, 2005, 2006). Interestingly, pathway assessment *in vivo* correlated better with ultimate treatment efficacy than *in vitro* signalling. In Detroit 562, the IHC changes were similar to those observed in the cell line Western blots. However, in HEP2 the signalling inhibition was less evident *in vivo* than *in vitro*, as little P70 inhibition was documented in the tumours. This may be reflective of a more complex environment, with multiple growth factors and stromal interactions that are not present *in vitro*. It is important though to bear in mind that none of these techniques is fully quantitative and both have a degree of subjectivity. A combination of three IHC markers was found to be an accurate early predictor of such antitumor activity. This may have implications in targeted therapy development, as new strategies consisting in sequential tumour sampling can be implemented to predict earlier which patients may obtain a benefit, and whom may need additional therapies to control their tumours. This is particularly relevant in SCC of the head and neck or cervix, where biopsies of the primary tumour require invasive procedures not free of potentially severe complications. On the other hand, FNA-based acquisition of tumour material from nodal origin is feasible and safe, as is usually coupled to ultrasound guidance and the quality and accuracy of the procedure can be tested on site by cytopathology personnel.

It is known that mTOR inhibitors work, at least in part, by decreasing angiogenesis (Bruns *et al*, 2004; Stephan *et al*, 2004). Although the efficacy of mTOR inhibition and an antiangiogenic effect have already been reported in similar SCC models (Amornphimoltham *et al*, 2005), the authors presented no direct link between these two phenomena, and hypothesised that the decrease in the vascular network may represent a direct consequence of the rapid reduction in the tumour size rather than being the cause of such antitumor effect. In the presented work, an upregulation of HIF-1*α* in tumours over time as their size increased was observed, that may be secondary to an increased hypoxia with tumour growth. In our model only dual EGFR and mTOR inhibition was capable of preventing HIF-1*α* upregulation *in vivo*. It has been suggested that HIF-1*α* is modulated by two afferent arms, one from the EGFR pathway and other from the PI3K/Akt pathway (Phillips *et al*, 2005), and we hypothesise that only a simultaneous inhibition of both afferent arms was able to abrogate HIF-1*α*. However, this observation needs to be cautiously interpreted, as although HIF-1*α* downregulation was seen in both combined arms, only in Detroit 562 there was decreased angiogenesis. Detroit 562 tumours expressed more HIF-1*α* at baseline, and also had a higher degree of increase over time with tumour growth, as seen by the 400 *vs* 200% increases over 7 days in Detroit 562 *vs* HEP2 tumours. This may indicate more dependence on HIF-1*α* to sustain vessels growth, and thus may explain the higher sensitivity to mTOR inhibition. Currently we do not have an explanation for the induction of HIF-1*α* in the EGFR-resistant cell line treated with erlotinib. Overexpression of proangiogenic factors has been shown to be an acquired mechanism of resistance of A431 squamous cell line to anti-EGFR therapies (Viloria-Petit *et al*, 2001; Viloria-Petit and Kerbel, 2004).

Per standard microvessel assessment, only the combination of erlotinib and temsirolimus in Detroit 562 induced inhibition of angiogenesis. Vessel growth followed a centripetal pattern from the tumour capsule, likely by recruiting murine stromal cellular components, and thus was an adequate model to test the impact of angiogenesis inhibition. We applied a novel method that examines the tumours in their entirety and that allowed us to examine this external invasive pattern of vessel formation; this novel approach correlated well with standard MVD. After breaking the blind of the analysed samples, it was found that temsirolimus, both alone but to a greater extent in combination with erlotinib, was able to inhibit capillary ingrowth in Detroit 562 xenografts but not in HEP2 tumours. The fact that despite this absence of antiangiogenic effect HEP2 tumours treated with temsirolimus had a significant growth decrement suggests that mTOR inhibitors work by multiple mechanisms as previously described. It also implies that the antitumor effect documented in Detroit 562 xenografts may also be related to mechanisms other than angiogenesis alone.

Cancer is the result of numerous alterations key cellular pathways and genes (Sjoblom *et al*, 2006), and blocking several of these may be necessary to achieve an antitumor effect. A reflection of this is the success of relatively nonspecific agents such as sorafenib or of agents that interfere at multiple levels such as bortezomib. Some authors advocate the development of ‘magic shotguns’ rather than ‘magic bullets’ as a more realistic and potentially successful approach to tackle a disease of such complexity as cancer (Mencher and Wang, 2005). Combining targeted agents is another rational strategy capable to induce tumour regressions in tumour models, as previously reported (Jimeno *et al*, 2005).

In summary, mTOR inhibitors showed antitumor activity in EGFR-resistant SCC cell lines. This effect was in part mediated by a decrease in angiogenesis. The combination with an EGFR inhibitor induced an additive effect in one of the models. Assessing pharmacodynamic markers by FNA-mediated biopsies early in therapy was predictive of outcome, showing that simultaneously inhibiting several pathways was required to induce tumour regression. This work represents state-of-the-art targeted therapy considering the recent approval of EGFR inhibitors in the corresponding patient population, and addresses the unmet need of primary or acquired resistance to such therapies.
